# Supplementary material for: Examining national health insurance fund members’ preferences and trade-offs for the attributes of contracted outpatient facilities in Kenya: A discrete choice experiment
Source: PLOS Glob Public Health. 2025 Apr 28;5(4):e0003557. doi: 10.1371/journal.pgph.0003557 (PMC12036850; doi:10.1371/journal.pgph.0003557)
Supplement: S1 File – — (DOCX) [file pgph.0003557.s001.docx]

| **Attributes** | **Coefficient** | **Standard error** | **z** | **P>z** | **[95% Confidence Interval** | |
| --- | --- | --- | --- | --- | --- | --- |
|  |  |  |  |  | **Lower** | **Upper** |
| **Availability of drugs** |  |  |  |  |  |  |
| Not always available | **Ref. (0)** |  |  |  |  |  |
| Always available | 2.55 | 0.17 | 14.97 | <0.001 | 2.22 | 2.89 |
| **Distance from household to facility** | -0.08 | 0.05 | -1.65 | 0.10 | -0.19 | 0.02 |
| **Waiting time at the facility until consultation** | -0.12 | 0.13 | -0.90 | 0.37 | -0.38 | 0.14 |
| **Attitude of health workers** |  |  |  |  |  |  |
| Health worker is harsh and abusive | **Ref. (0)** |  |  |  |  |  |
| Health worker is respectful | 0.36 | 0.16 | 2.19 | 0.03 | 0.04 | 0.68 |
| **Cleanliness of the facility** |  |  |  |  |  |  |
| Facility (toilets-rooms-floors) are not always clean | **Ref. (0)** |  |  |  |  |  |
| Facility (toilets-rooms-floors) are always clean | 0.16 | 0.16 | 0.97 | 0.33 | -0.16 | 0.48 |
| **Cadre of health worker seen during a consultation** |  |  |  |  |  |  |
| Nurse | **Ref. (0)** |  |  |  |  |  |
| Clinical Officer | 0.79 | 0.20 | 3.91 | <0.001 | 0.39 | 1.19 |
| Medical Doctor | 1.31 | 0.21 | 6.28 | <0.001 | 0.90 | 1.72 |
| **Opening hours of the facility** |  |  |  |  |  |  |
| 12 hours excluding weekend | **Ref. (0)** |  |  |  |  |  |
| 12 hours including weekends | 0.19 | 0.25 | 0.78 | 0.44 | -0.30 | 0.69 |
| 24 hours excluding weekends | -0.05 | 0.27 | -0.20 | 0.84 | -0.58 | 0.47 |
| 24 hours including weekends | 0.37 | 0.25 | 1.50 | 0.13 | -0.11 | 0.86 |
| **Model fit statistics** |  |  |  |  |  |  |
| Log-likelihood (final) | -436.381 | | | | | |
| Number of Observations | 912 | | | | | |
| Akaike Information Criteria (AIC) | 892.761 | | | | | |
| Bayesian Information Criteria (BIC) | 940.918 | | | | | |
