# Supplementary material for: Examining national health insurance fund members’ preferences and trade-offs for the attributes of contracted outpatient facilities in Kenya: A discrete choice experiment
Source: PLOS Glob Public Health. 2025 Apr 28;5(4):e0003557. doi: 10.1371/journal.pgph.0003557 (PMC12036850; doi:10.1371/journal.pgph.0003557)
Supplement: S2 File – — (DOCX) [file pgph.0003557.s002.docx]

Summary of key differences between Nurses, Clinical Officers, and Medical Doctors

| **Aspect** | **Nurses** | **Clinical Officers** | **Medical Doctors** |
| --- | --- | --- | --- |
| Education | Diploma or Bachelor’s degree in Nursing | Diploma or Bachelor’s degree in clinical medicine | Bachelor's degree in medicine and surgery |
| Patient care | Patient care, health promotion, prevention. However, they also do diagnosis and treatment in lower-level facilities | Diagnosis, treatment, and minor surgical skills | Diagnosis, treatment, and major surgical skills |
