# Supplementary material for: Examining national health insurance fund members’ preferences and trade-offs for the attributes of contracted outpatient facilities in Kenya: A discrete choice experiment
Source: PLOS Glob Public Health. 2025 Apr 28;5(4):e0003557. doi: 10.1371/journal.pgph.0003557 (PMC12036850; doi:10.1371/journal.pgph.0003557)
Supplement: S3 File – — (DOCX) [file pgph.0003557.s003.docx]

**A CHOICE EXPERIMENT TO ELICIT THE PREFERENCES OF NHIF MEMBERS FOR THE ATTRIBUTES OF NHIF-CONTRACTED OUTPATIENT FACILITIES IN KENYA.**

**Section A. Information about Area** *(To be completed by researcher)*

Questionnaire id: ____________________ Survey Date: ____________________

Survey time: ________________________ County: ________________________

Sub county: _________________________ Interviewer code: _______________

**Section B. Information about you** *(To be completed by the respondent)*

1. What is your gender? (*Tick one of the boxes below.)*

Male  Female

1. How old are you? (*Provide age in years*) _____________
2. What is your employment status? (*Tick one of the boxes below.)*

Not employed

Employed in the **Informal** sector (e.g., self-employed)

Employed in the **Formal** sector (i.e., on a payroll)

1. How far do you live from your household to the nearest NHIF-contracted outpatient facility (in Kilometres)? ____________
2. How long does it take you to get to an NHIF-contracted facility? ____________
3. What means of transport do you use when travelling to the facility?

Walking

Boda Boda

Tuktuk

Public transport (Matatu or Bus)

Private car

Other (Specify) ________________________________________________

1. Do you know that NHIF requires you to choose and register at an outpatient facility before you can use outpatient services?

Yes

No

1. If Yes to the above, through which channel did you learn about the requirement for you to choose an outpatient facility before you can get outpatient services under NHIF?

NHIF Branch Office

NHIF Website

NHIF social media (Facebook etc.)

Media (e.g., Newspaper, Television or Radio)

Other (Specify)_____________________________________________________

**Section C. Choice experiment** *(For the respondent to read – Researcher can describe)*

Imagine you are to choose a facility where you can access outpatient service under NHIF. You will be presented with 12 sets of tasks, each representing two alternatives defining the characteristics of a possible health facility. These alternatives differ only by the levels of the following six characteristics:

1. **Availability of drugs** – Whether drugs would always be available at the facility, or they are not always available (you have to go buy these elsewhere sometimes).
2. **Distance from household to the facility**– Whether the facility is located within 1 Kilometre or 3 Kilometres or 5 Kilometres to your homestead.
3. **Waiting time at the facility for consultation**. This is the time you will have to wait in a queue once you arrive at the facility to when you can get a consultation. This may be up to 1 hour or 2 hours or 3 hours.
4. **Attitude of health worker**. This refers to the way a health worker speaks to you at the facility. Whether the health worker is harsh and abusive or the health worker speaks and treats you with respect.
5. **Cleanliness of the facility.** This refers to whether the facility you visit has its toilets, rooms and floors always clean or the toilets, rooms and floors are not always clean.
6. **Cadre of healthcare worker you see at the facility.** This refers to who you would like to see during consultation at the facility. Whether a nurse or clinical officer or a medical doctor.

For each task, you will be asked to choose, by ticking, whether you would select health facility A or health facility B.

**Section D. Choice experiment tasks** *(For the respondent to read – Researcher can describe)*

**Practice Scenario 1** *(Researcher to use this as a demonstration)*

|  | **Health Facility A** | **Health Facility B** |
| --- | --- | --- |
| **Availability of Drugs** | Drugs Not Always available | Drugs Not Always available |
| **Distance from household to the facility (Kilometres)** | 3 Kilometre | 5 Kilometres |
| **Waiting time at facility for consultation (Hours)** | 2 Hour | 3 Hours |
| **Attitude of health worker** | Health worker is respectful | Health worker is harsh and abusive |
| **Cleanliness of the facility** | Facility (toilets-rooms-floors) always clean | Facility (toilets-rooms-floors) NOT always clean |
| **Cadre of health worker you see for consultation** | Medical Doctor | Clinical officer |
| **Which facility would you choose? (Tick one only)** |  |  |
|  |  |  |

**Practice Scenario 2** *(To be completed by respondent)*

|  | **Health Facility A** | **Health Facility B** |
| --- | --- | --- |
| **Availability of Drugs** | Drugs Always available | Drugs NOT always available |
| **Distance from household to the facility (Kilometres)** | 1 Kilometre | 5 Kilometres |
| **Waiting time at facility for consultation (Hours)** | 1 Hour | 3 Hours |
| **Attitude of health worker** | Health worker is respectful | Health worker is harsh and abusive |
| **Cleanliness of the facility** | Facility (toilets-rooms-floors) always clean | Facility (toilets-rooms-floors) NOT always clean |
| **Cadre of health worker you see for consultation** | Medical Doctor | Nurse |
| **Which facility would you choose? (Tick one only)** |  |  |
|  |  |  |

**Practice Scenario 3** *(To be completed by respondent)*

|  | **Health Facility A** | **Health Facility B** |
| --- | --- | --- |
| **Availability of Drugs** | Drugs Always available | Drugs NOT always available |
| **Distance from household to the facility (Kilometres)** | 5 Kilometres | 1 Kilometre |
| **Waiting time at facility for consultation (Hours)** | 3 Hours | 1 Hour |
| **Attitude of health worker** | Health worker is harsh and abusive | Health worker is respectful |
| **Cleanliness of the facility** | Facility (toilets-rooms-floors) always clean | Facility (toilets-rooms-floors) NOT always clean |
| **Cadre of health worker you see for consultation** | Nurse | Clinical Officer |
| **Which facility would you choose? (Tick one only)** |  |  |
|  |  |  |

**Scenario 1** *(To be completed by respondent)*

|  | **Health Facility A** | **Health Facility B** |
| --- | --- | --- |
| **Availability of Drugs** | Drugs Not Always available | Drugs Not Always available |
| **Distance from household to the facility (Kilometres)** | 1 Kilometre | 5 Kilometres |
| **Waiting time at facility for consultation (Hours)** | 1 Hour | 3 Hours |
| **Attitude of health worker** | Health worker is harsh and abusive | Health worker is respectful |
| **Cleanliness of the facility** | Facility (toilets-rooms-floors) always clean | Facility (toilets-rooms-floors) NOT always clean |
| **Cadre of health worker you see for consultation** | Medical Doctor | Clinical officer |
| **Which facility would you choose? (Tick one only)** |  |  |
|  |  |  |

**Scenario 2** *(To be completed by respondent)*

|  | **Health Facility A** | **Health Facility B** |
| --- | --- | --- |
| **Availability of Drugs** | Drugs Always available | Drugs Not Always available |
| **Distance from household to the facility (Kilometres)** | 5 Kilometres | 1 Kilometre |
| **Waiting time at facility for consultation (Hours)** | 2 Hours | 2 Hours |
| **Attitude of health worker** | Health worker is respectful | Health worker is harsh and abusive |
| **Cleanliness of the facility** | Facility (toilets-rooms-floors) always clean | Facility (toilets-rooms-floors) NOT always clean |
| **Cadre of health worker you see for consultation** | Nurse | Medical Doctor |
| **Which facility would you choose? (Tick one only)** |  |  |
|  |  |  |

**Scenario 3** *(To be completed by respondent)*

|  | **Health Facility A** | **Health Facility B** |
| --- | --- | --- |
| **Availability of Drugs** | Drugs Not Always available | Drugs Always available |
| **Distance from household to the facility (Kilometres)** | 5 Kilometres | 1 Kilometre |
| **Waiting time at facility for consultation (Hours)** | 3 Hours | 1 Hour |
| **Attitude of health worker** | Health worker is respectful | Health worker is harsh and abusive |
| **Cleanliness of the facility** | Facility (toilets-rooms-floors) always clean | Facility (toilets-rooms-floors) NOT always clean |
| **Cadre of health worker you see for consultation** | Medical Doctor | Clinical officer |
| **Which facility would you choose? (Tick one only)** |  |  |
|  |  |  |

**Scenario 4** *(To be completed by respondent)*

|  | **Health Facility A** | **Health Facility B** |
| --- | --- | --- |
| **Availability of Drugs** | Drugs Always available | Drugs Always available |
| **Distance from household to the facility (Kilometres)** | 1 Kilometre | 5 Kilometres |
| **Waiting time at facility for consultation (Hours)** | 3 Hours | 1 Hour |
| **Attitude of health worker** | Health worker is respectful | Health worker is harsh and abusive |
| **Cleanliness of the facility** | Facility (toilets-rooms-floors) NOT always clean | Facility (toilets-rooms-floors) always clean |
| **Cadre of health worker you see for consultation** | Nurse | Medical Doctor |
| **Which facility would you choose? (Tick one only)** |  |  |
|  |  |  |

**Scenario 5** *(To be completed by respondent)*

|  | **Health Facility A** | **Health Facility B** |
| --- | --- | --- |
| **Availability of Drugs** | Drugs Always available | Drugs Not Always available |
| **Distance from household to the facility (Kilometres)** | 3 Kilometres | 3 Kilometres |
| **Waiting time at facility for consultation (Hours)** | 2 Hours | 2 Hours |
| **Attitude of health worker** | Health worker is harsh and abusive | Health worker is respectful |
| **Cleanliness of the facility** | Facility (toilets-rooms-floors) NOT always clean | Facility (toilets-rooms-floors) always clean |
| **Cadre of health worker you see for consultation** | Clinical Officer | Nurse |
| **Which facility would you choose? (Tick one only)** |  |  |
|  |  |  |

**Scenario 6** *(To be completed by respondent)*

|  | **Health Facility A** | **Health Facility B** |
| --- | --- | --- |
| **Availability of Drugs** | Drugs Always available | Drugs Not Always available |
| **Distance from household to the facility (Kilometres)** | 3 Kilometres | 3 Kilometres |
| **Waiting time at facility for consultation (Hours)** | 2 Hours | 2 Hours |
| **Attitude of health worker** | Health worker is respectful | Health worker is harsh and abusive |
| **Cleanliness of the facility** | Facility (toilets-rooms-floors) NOT always clean | Facility (toilets-rooms-floors) always clean |
| **Cadre of health worker you see for consultation** | Nurse | Clinical officer |
| **Which facility would you choose? (Tick one only)** |  |  |
|  |  |  |

**Scenario 7** *(To be completed by respondent)*

|  | **Health Facility A** | **Health Facility B** |
| --- | --- | --- |
| **Availability of Drugs** | Drugs Not Always available | Drugs Always available |
| **Distance from household to the facility (Kilometres)** | 3 Kilometres | 3 Kilometres |
| **Waiting time at facility for consultation (Hours)** | 1 Hour | 3 Hours |
| **Attitude of health worker** | Health worker is respectful | Health worker is harsh and abusive |
| **Cleanliness of the facility** | Facility (toilets-rooms-floors) NOT always clean | Facility (toilets-rooms-floors) always clean |
| **Cadre of health worker you see for consultation** | Clinical Officer | Nurse |
| **Which facility would you choose? (Tick one only)** |  |  |
|  |  |  |

**Scenario 8** *(To be completed by respondent)*

|  | **Health Facility A** | **Health Facility B** |
| --- | --- | --- |
| **Availability of Drugs** | Drugs Not Always available | Drugs Not Always available |
| **Distance from household to the facility (Kilometres)** | 5 Kilometres | 1 Kilometre |
| **Waiting time at facility for consultation (Hours)** | 3 Hours | 1 Hour |
| **Attitude of health worker** | Health worker is harsh and abusive | Health worker is respectful |
| **Cleanliness of the facility** | Facility (toilets-rooms-floors) NOT always clean | Facility (toilets-rooms-floors) always clean |
| **Cadre of health worker you see for consultation** | Medical Doctor | Nurse |
| **Which facility would you choose? (Tick one only)** |  |  |
|  |  |  |

**Scenario 9** *(To be completed by respondent)*

|  | **Health Facility A** | **Health Facility B** |
| --- | --- | --- |
| **Availability of Drugs** | Drugs Always available | Drugs Always available |
| **Distance from household to the facility (Kilometres)** | 3 Kilometres | 3 Kilometres |
| **Waiting time at facility for consultation (Hours)** | 1 Hour | 3 Hours |
| **Attitude of health worker** | Health worker is harsh and abusive | Health worker is respectful |
| **Cleanliness of the facility** | Facility (toilets-rooms-floors) NOT always clean | Facility (toilets-rooms-floors) always clean |
| **Cadre of health worker you see for consultation** | Nurse | Clinical officer |
| **Which facility would you choose? (Tick one only)** |  |  |
|  |  |  |

**Scenario 10** *(To be completed by respondent)*

|  | **Health Facility A** | **Health Facility B** |
| --- | --- | --- |
| **Availability of Drugs** | Drugs Always available | Drugs Always available |
| **Distance from household to the facility (Kilometres)** | 1 Kilometre | 5 Kilometres |
| **Waiting time at facility for consultation (Hours)** | 3 Hours | 1 Hour |
| **Attitude of health worker** | Health worker is harsh and abusive | Health worker is respectful |
| **Cleanliness of the facility** | Facility (toilets-rooms-floors) always clean | Facility (toilets-rooms-floors) NOT always clean |
| **Cadre of health worker you see for consultation** | Clinical Officer | Medical Doctor |
| **Which facility would you choose? (Tick one only)** |  |  |
|  |  |  |

**Scenario 11** *(To be completed by respondent)*

|  | **Health Facility A** | **Health Facility B** |
| --- | --- | --- |
| **Availability of Drugs** | Drugs Not Always available | Drugs Always available |
| **Distance from household to the facility (Kilometres)** | 1 Kilometre | 5 Kilometres |
| **Waiting time at facility for consultation (Hours)** | 2 Hours | 2 Hours |
| **Attitude of health worker** | Health worker is respectful | Health worker is harsh and abusive |
| **Cleanliness of the facility** | Facility (toilets-rooms-floors) always clean | Facility (toilets-rooms-floors) NOT always clean |
| **Cadre of health worker you see for consultation** | Medical Doctor | Nurse |
| **Which facility would you choose? (Tick one only)** |  |  |
|  |  |  |

**Scenario 12** *(To be completed by respondent)*

|  | **Health Facility A** | **Health Facility B** |
| --- | --- | --- |
| **Availability of Drugs** | Drugs Not Always available | Drugs Not Always available |
| **Distance from household to the facility (Kilometres)** | 5 Kilometres | 1 Kilometre |
| **Waiting time at facility for consultation (Hours)** | 1 Hour | 3 Hours |
| **Attitude of health worker** | Health worker is harsh and abusive | Health worker is respectful |
| **Cleanliness of the facility** | Facility (toilets-rooms-floors) always clean | Facility (toilets-rooms-floors) NOT always clean |
| **Cadre of health worker you see for consultation** | Clinical Officer | Medical Doctor |
| **Which facility would you choose? (Tick one only)** |  |  |
|  |  |  |

**Section E. Supplementary information** *(To be completed by the respondent)*

1. Do you have any chronic conditions?

Yes No

1. If yes to the above, what is the severity of your condition?

Very severe Severe Moderate

1. Do you like that you can choose a facility of your choice? *(Tick one of the boxes below)*

Yes No

1. Have you already chosen an NHIF-contracted outpatient facility?

Yes No

1. If yes to the above:
   1. What is the availability of drugs in the facility you selected?

Always Available

Not always available

- 1. What is the distance to this facility (in Kilometres)?
  2. How long does it take to get to the facility?
  3. What means of transport do you use to get to the facility?

Walking

Boda Boda

Tuktuk

Public transport (Matatu or Bus)

Private car

Other (Specify) __________________________________

- 1. What is the waiting time while at the facility when you arrive to when you get consultation on average (time in hours)?
  2. How is the attitude of the health workers in the facility you selected?

Always talked to well and respected

Health worker talks harshly or with abuses

- 1. How is the cleanliness of the facility you selected?

Facility (toilets-rooms-floors) always clean

Facility (toilets-rooms-floors) NOT always clean

- 1. Which cadre of health worker do you see for consultation at the facility?

Nurse

Clinical Officer

Medical Doctor

_________________________________________________________________

**END (Thank you for your time)**
