# Supplementary material for: Examining national health insurance fund members’ preferences and trade-offs for the attributes of contracted outpatient facilities in Kenya: A discrete choice experiment
Source: PLOS Glob Public Health. 2025 Apr 28;5(4):e0003557. doi: 10.1371/journal.pgph.0003557 (PMC12036850; doi:10.1371/journal.pgph.0003557)
Supplement: S4 File – — (DOCX) [file pgph.0003557.s004.docx]

LCA results

| **Attributes** | **Class 1 - Attitude-focused class** | | | **Class 2 - Cadre-focused class** | | | **Class 3 - Drug-focused class** | | |
| --- | --- | --- | --- | --- | --- | --- | --- | --- | --- |
|  | **Coefficient** | **95% CI** | **P-value** | **Coefficient** | **95% CI** | **P-value** | **Coefficient** | **95% CI** | **P-value** |
| **Availability of drugs** |  |  |  |  |  |  |  |  |  |
| Not always available | Ref. (0) |  |  | Ref. (0) |  |  | Ref. (0) |  |  |
| Always available | 0.534 | -0.333 to 1.401 | 0.228 | 0.869 | 0.437 to 1.301 | <0.001 | 1.553 | 1.367 to 1.738 | <0.001 |
| **Distance from household to facility** | -0.311 | -0.492 to -0.131 | 0.001 | -0.044 | -0.116 to 0.028 | 0.227 | -0.055 | -0.081 to -0.029 | <0.001 |
| **Waiting time at the facility until consultation** | -0.261 | -0.529 to 0.006 | 0.055 | -0.31 | -0.464 to -0.156 | <0.001 | -0.167 | -0.219 to -0.114 | <0.001 |
| **Attitude of health workers** |  |  |  |  |  |  |  |  |  |
| Health worker is harsh and abusive | Ref. (0) |  |  | Ref. (0) |  |  | Ref. (0) |  |  |
| Health worker is respectful | 3.516 | 2.606 to 4.427 | <0.001 | 0.836 | 0.522 to 1.150 | <0.001 | 0.731 | 0.620 to 0.843 | <0.001 |
| **Cleanliness of the facility** |  |  |  |  |  |  |  |  |  |
| Facility (toilets-rooms-floors) are not always clean | Ref. (0) |  |  | Ref. (0) |  |  | Ref. (0) |  |  |
| Facility (toilets-rooms-floors) are always clean | 0.566 | 0.068 to 1.063 | 0.026 | 0.478 | 0.179 to 0.777 | 0.002 | 0.677 | 0.574 to 0.781 | <0.001 |
| **Cadre of health worker seen during a consultation** |  |  |  |  |  |  |  |  |  |
| Nurse | Ref. (0) |  |  | Ref. (0) |  |  | Ref. (0) |  |  |
| Clinical Officer | -0.615 | -1.287 to 0.058 | 0.073 | 2.081 | 1.573 to 2.588 | <0.001 | 0.165 | 0.031 to 0.299 | 0.016 |
| Medical Doctor | -0.032 | -1.035 to 0.971 | 0.950 | 3.486 | 2.868 to 4.105 | <0.001 | 0.847 | 0.672 to 1.021 | <0.001 |
| **Class share** | 16.8% | | | 19.8% | | | 63.4% | | |
